# Reply to Ross, K. Comment on “Polak et al. Principles and Limitations of miRNA Purification and Analysis in Whole Blood Collected during Ablation Procedure from Patients with Atrial Fibrillation. *J. Clin. Med.* 2024, *13*, 1898”

**DOI:** 10.3390/jcm13133745

**Published:** 2024-06-26

**Authors:** Mateusz Polak, Joanna Wieczorek, Malwina Botor, Aleksandra Auguścik-Duma, Andrzej Hoffmann, Anna Wnuk-Wojnar, Katarzyna Gawron, Katarzyna Mizia-Stec

**Affiliations:** 1 First Department of Cardiology, School of Medicine in Katowice, Medical University of Silesia, 40-055 Katowice, Poland; 2 Department of Molecular Biology and Genetics, School of Medicine in Katowice, Medical University of Silesia, 40-055 Katowice, Poland

Thank you for your interest and insightful comments on our article [1]. We appreciate your detailed analysis and valuable suggestions [2], which undoubtedly will contribute to enhancing future research in this area.

As mentioned in our article, we conducted optimization studies using various kits for miRNA isolation from blood, adhering strictly to each manufacturer’s protocol. Consequently, the elution volumes were as follows: 14 µL for the miRNeasy Serum/Plasma Kit, 20 µL for the miRNeasy Serum/Plasma Advanced Kit, and 2 × 40 µL for the PAXgene RNA Blood Kit (all from Qiagen, Hilden, Germany). We agree that including the final total RNA yield isolated would be beneficial for readers. However, we chose the RNA concentration because it provides insights into both the quality and quantity of RNA yielded by each method.

Considering the comment on AF miRNA profile comparison with the control, we presume that the small number of replicates and biological material used in our study would not yield statistically reliable results for miRNA detection in plasma. However, we agree that this is a promising direction for future research once the method is further optimized. Additionally, we acknowledge that our current work primarily establishes technical validity rather than biological relevance, but the key to successful research is precise optimization of the technical process.

While next-generation sequencing (NGS) approaches are certainly worth considering, we opted for the PCR array due to its easier availability in laboratories and lower cost, but we certainly agree that NGS approaches are more accurate.

Finally, we completely agree that circular RNAs (circRNAs), which are increasingly being linked to the pathogenesis of AF, warrant detailed analysis [3,4,5]. Additionally, exploring the interactions among specified circRNAs and miRNAs in AF would be of particular interest. The role of non-coding RNA molecules and their interactions and impact on the pathogenesis of AF is of interest for further research in our laboratory.
